# Correlation of TNF-α polymorphisms with susceptibility to lung cancer: evidence from a meta-analysis based on 29 studies

**DOI:** 10.1186/s12885-024-12854-x

**Published:** 2024-09-06

**Authors:** Seyed Masoud HaghighiKian, Ahmad Shirinzadeh-Dastgiri, Reza Ershadi, Mohammad Vakili-Ojarood, Maedeh Barahman, Seyed Alireza Dastgheib, Fatemeh Asadian, Amirmasoud Shiri, Maryam Aghasipour, Amirhossein Rahmani, Kazem Aghili, Hossein Neamatzadeh

**Affiliations:** 1 Department of General Surgery, School of Medicine Hazrat-e Rasool General Hospital, Iran University of Medical Sciences, Tehran, Iran; 2 Department of Surgery, School of Medicine, Shohadaye Haft-e Tir Hospital, Iran University of Medical Sciences, Tehran, Iran; 3 Department of Thoracic Surgery, Imam Khomeini Hospital Complex, Tehran University of Medical Sciences, Tehran, Iran; 4 Department of Surgery, School of Medicine, Ardabil University of Medical Sciences, Ardabil, Iran; 5 Department of Radiation Oncology, Firoozgar Clinical Research Development Center (FCRDC), Firoozgar Hospital, Iran University of Medical Sciences, Tehran, Iran; 6 Department of Medical Genetics, School of Medicine, Shiraz University of Medical Sciences, Shiraz, Iran; 7 Department of Medical Laboratory Sciences, School of Paramedical Science, Shiraz University of Medical Sciences, Shiraz, Iran; 8 School of Medicine, General Practitioner, Shiraz University of Medical Sciences, Shiraz, Iran; 9 Department of Cancer Biology, College of Medicine, University of Cincinnati, Cincinnati, OH USA; 10 Department of Plastic Surgery, Iranshahr University of Medical Sciences, Iranshahr, Iran; 11 Department of Radiology, School of Medicine, Shahid Rahnamoun Hospital, Shahid Sadoughi University of Medical Sciences, Yazd, Iran; 12 Mother and Newborn Health Research Center, Shahid Sadoughi University of Medical Sciences, Yazd, Iran

**Keywords:** Lung cancer, TNF-α, Polymorphisms, Meta-analysis, Susceptibility, Ethnicity

## Abstract

**Objective:**

This meta-analysis aims to clarify the association between the TNF-α -308G > A and − 238G > A polymorphisms and lung cancer risk.

**Method:**

A comprehensive search was conducted for relevant articles across databases such as PubMed, Google Scholar, Web of Science, EMBASE, and CNKI, up to September 25, 2023. Lung cancer risk was assessed by calculating odds ratios (ORs) and their 95% confidence intervals (CIs). The Z-test was used to determine the significance of combined ORs, with *P* < 0.05 considered statistically significant. All analyses were performed using Comprehensive Meta-Analysis (CMA) 2.0 software.

**Results:**

The analysis included 19 case-control studies with 3,838 cases and 5,306 controls for the TNF-α -308G > A polymorphism, along with 10 studies comprising 2,427 cases and 2,357 controls for the − 238G > A polymorphism. The − 308G > A polymorphism showed no significant overall relationships, though in the Asian subgroup, the A allele was significantly reduced compared to G (OR: 0.831, *p* = 0.028) and the AA genotype showed significant reductions versus GG (OR: 0.571, *p* = 0.021), with no significant correlation in Caucasians. In non-small cell lung cancer (NSCLC), the A allele was associated with increased risk compared to G (OR: 1.131, *p* = 0.049). For the − 238G > A polymorphism, the AA genotype significantly increased risk compared to GG (OR: 3.171, *p* = 0.014), while showing a protective effect in Caucasians (OR: 0.120, *p* = 0.024) and a heightened risk in Asians (OR: 7.990, *p* = 0.007). In small cell lung cancer (SCLC), the A allele conferred protective effects, whereas NSCLC showed increased risk for the AA genotype (OR: 11.375, *p* = 0.002).

**Conclusion:**

The − 308G > A polymorphism has no significant overall relationships but suggests a protective role of the A allele in the Asian subgroup. Conversely, the − 238G > A polymorphism presents a complex risk profile, increasing lung cancer likelihood in Asians while protecting Caucasians. Notably, the AA genotype significantly raises risk for NSCLC, indicating its potential as a risk factor.

## Introduction

Lung cancer represents a significant challenge in global healthcare, being the second most prevalent malignancy and the foremost cause of cancer-related mortality among both men and women worldwide [1]. Despite recent improvements in survival rates for lung cancer patients, it remains the leading cause of cancer deaths globally, with only 20% of newly diagnosed patients surviving beyond five years [2]. The Global Cancer Incidence, Mortality, and Prevalence (GLOBOCAN) report of 2020 indicates that lung cancer is the second most common cancer and the leading cause of cancer deaths, with an estimated 1.8 million deaths, accounting for 18% of total cancer fatalities [3]. Lung cancer is classified into two primary types: small cell lung cancer (SCLC), which affects approximately 15 to 20% of patients, and non-small cell lung cancer (NSCLC), which constitutes 80–85% of cases globally [4]. NSCLC includes various malignancies, primarily adenocarcinoma (ADC), squamous cell carcinoma (SCC), and large cell carcinoma (LCC), with ADC and SCC representing over 70% of NSCLC instances [4, 5]. Although NSCLC subtypes share numerous biological characteristics, they differ in origin, location, and growth patterns, suggesting distinct molecular mechanisms underlying their development [6]. Tobacco smoking is the principal risk factor, responsible for 80 to 90% of all lung cancer diagnoses, with the incidence and mortality rates largely influenced by tobacco consumption patterns across different populations and time periods [7]. Additional risk factors for lung cancer include secondhand smoke exposure, genetic predisposition, poor dietary habits, a family history of lung cancer, certain vitamin deficiencies, and exposure to hazardous chemicals [7, 8].

Tumor necrosis factor-alpha (TNF-α) is a proinflammatory cytokine that is integral to various biological functions and is notably implicated in the pathogenesis of inflammatory, autoimmune, and malignancies [9, 10]. TNF-α mediates apoptosis through Caspase activation, operating directly or indirectly by promoting the release of apoptogenic factors from mitochondria across different cell types [11, 12]. Furthermore, TNF-α significantly contributes to angiogenesis by enhancing endothelial cell proliferation and elevating the expression of pro-angiogenic factors, such as basic fibroblast growth factor (bFGF), interleukin-8 (IL-8), and vascular endothelial growth factor (VEGF). It also stimulates the expression of adhesion molecules, including intracellular adhesion molecule (ICAM)-1, E-selectin, and vascular cell adhesion molecule (VCAM-1), thereby facilitating the invasion of metastatic tumor cells [13–15]. Due to its complex role as an oncogene and a potential tumor suppressor, TNF-α has gained considerable interest in contemporary cancer research. Specifically, polymorphisms in the TNF-α gene have been identified as risk factors for several cancers, including breast, gastric, and hepatocellular carcinomas, as evidenced by meta-analyses. The human TNF-α gene is located within the Class III region of the major histocompatibility complex (MHC) on chromosome 6 (6p21.31), encompassing approximately 3 kb and consisting of four exons [16, 17]. Notably, polymorphisms in the promoter region of the TNF-α gene have been characterized, including − 1031 T > C (rs1799964), -863 C > A (rs1800630), -857 C > T (rs1799724), -308G > A (rs1800629), and − 238G > A (rs361525). However, previous studies exploring the relationship between TNF-α polymorphisms and lung cancer risk among diverse ethnic groups have produced inconsistent results, with some findings indicating an association while others do not. To resolve the debate regarding the associations of TNF-α -308G > A and − 238G > A polymorphisms with lung cancer risk, we undertook this meta-analysis by identifying all relevant studies.

## Materials and methods

### Search strategy

This meta-analysis followed the Preferred Reporting Items for Systematic Reviews and Meta-Analyses (PRISMA) guidelines to enhance the transparency and completeness of findings from observational epidemiological studies. A systematic literature search was conducted across various electronic databases, including the Cochrane Library, PubMed, Google Scholar, EBSCOhost, EMBASE, Web of Science, the Islamic World Science Citation Center (ISC), the Scientific Information Database (SID), Wanfang Data, China National Knowledge Infrastructure (CNKI), Scopus, ClinicalTrials.gov, LILACS, CINAHL, arXiv, and the Directory of Open Access Journals (DOAJ), to ensure a comprehensive review of the relevant literature. The main objective of this comprehensive search was to identify studies examining the relationship between TNF-α polymorphisms and lung cancer incidence, incorporating research up to September 25, 2023, to include the latest findings. A carefully selected set of keywords was used, including “lung neoplasms,” “lung carcinomas,” “lung adenocarcinoma,” “lung cancer,” “small cell lung cancer,” “non-small cell lung cancer,” “squamous cell carcinoma,” “adenocarcinoma,” “large cell carcinoma,” “tumor necrosis factor-alpha,” “TNF-α,” “cachexin,” “cachectin,” and specific variants like “TNF-α -308G > A” and “rs1800629.” Additional keywords were “TNF-α -238G > A,” “rs361525,” “cytokine,” “inflammatory gene,” “single nucleotide polymorphism (SNP),” “genetic polymorphism,” “lung tumor markers,” “risk factors for lung cancer,” “molecular epidemiology of lung cancer,” and “genetic susceptibility to lung cancer.” To improve the search for relevant studies, researchers manually examined the reference lists of identified articles, such as reviews and meta-analyses. This careful approach aimed to reduce the chance of missing important research by identifying pertinent articles not found in the initial electronic search. Incorporating these additional findings enhanced the analysis’s comprehensiveness and robustness.

### Inclusion and exclusion criteria

Eligible investigations were included based on the following criteria: (1) case-control or cohort design; (2) assessment of the association between TNF-α -308G > A and − 238G > A polymorphisms and lung cancer risk; (3) adequate genotypic data for both cases and controls; (4) reported risk estimates as relative risk (RR) and/or odds ratio (OR) with a 95% confidence interval (CI), or sufficient data to calculate OR with 95% CI; (5) involvement of human subjects; (6) availability of full-text articles; and (7) population-based or hospital-based case-control studies. Studies were excluded if they: (1) involved animal or in vitro models; (2) focused on sibling, twin, family-based, or linkage investigations; (3) provided insufficient published data; (4) were case reports, comments, posters, reviews, letters to editors, editorials, or conference abstracts; and (5) presented duplicated or overlapping data. The meta-analysis imposed no language restrictions.

### Date extraction

Two authors independently extracted data and assessed quality, reaching consensus on all search terms used in the study. Selected studies underwent a rigorous review based on defined inclusion and exclusion criteria to ensure the relevance of the extracted information. In case of disagreement, detailed discussions involving a third author were conducted to reach unanimous resolutions. When further information or raw data were needed, the authors contacted the corresponding authors of the studies. A comprehensive dataset was compiled, including essential variables such as the first author’s name, publication year, country of origin, and participant ethnicity (Caucasian, Asian, African, and Mixed backgrounds). Controls were categorized as HB or PB, with genotyping methods and the numbers of cases and controls fully documented. The analysis focused on the genotypic distributions of the TNF-α -308G > A and − 238G > A polymorphisms in both cases and controls, also assessing the minor allele frequency (MAF) in healthy individuals. Additionally, Hardy-Weinberg equilibrium (HWE) test probabilities were calculated for these polymorphisms in the control group, providing insights into genetic distribution and potential biases. This thorough evaluation enhances the reliability and validity of the findings.

### Statistical analysis

This meta-analysis assessed the relationship between TNF-α polymorphisms and lung cancer risk by calculating ORs and 95% CIs. The significance of pooled ORs was evaluated using the Z-test, with a threshold of *P* < 0.05. Five genetic models were applied for each polymorphism: allele (B vs. A), homozygote (BB vs. AA), heterozygote (BA vs. AA), dominant (BB + BA vs. AA), and recessive (BB vs. BA + AA) to thoroughly assess disease risk. Heterogeneity among studies was assessed using the Q-test (*P* < 0.01) and quantified with the I² statistic, where values over 50% indicated significant heterogeneity. A fixed-effects model (Mantel-Haenszel method) was used for pooled OR calculations in the absence of heterogeneity, while a random-effects model (DerSimonian and Laird method) was applied when significant heterogeneity was present. The chi-square test assessed deviations from HWE with a significance criterion of *P* < 0.05. Stratified analyses by ethnicity, genotyping technique, and control sources further clarified associations. Robustness was verified through sensitivity analyses, excluding individual studies and those violating HWE (*P* < 0.05). To evaluate publication bias, Egger’s linear regression test and Begg’s funnel plots were employed, with plot symmetry analyzed using Egger’s test, where *P* < 0.05 indicated significant bias. These methodologies allowed for a comprehensive assessment of the relationship between TNF-α polymorphisms and lung cancer risk. All analyses were conducted using Comprehensive Meta-Analysis (CMA) 2.0 software (Biostat, USA), known for its robustness in meta-analytic methodologies, with statistical significance defined by two-sided P-values less than 0.05.

## Results

**Fig. 1:**
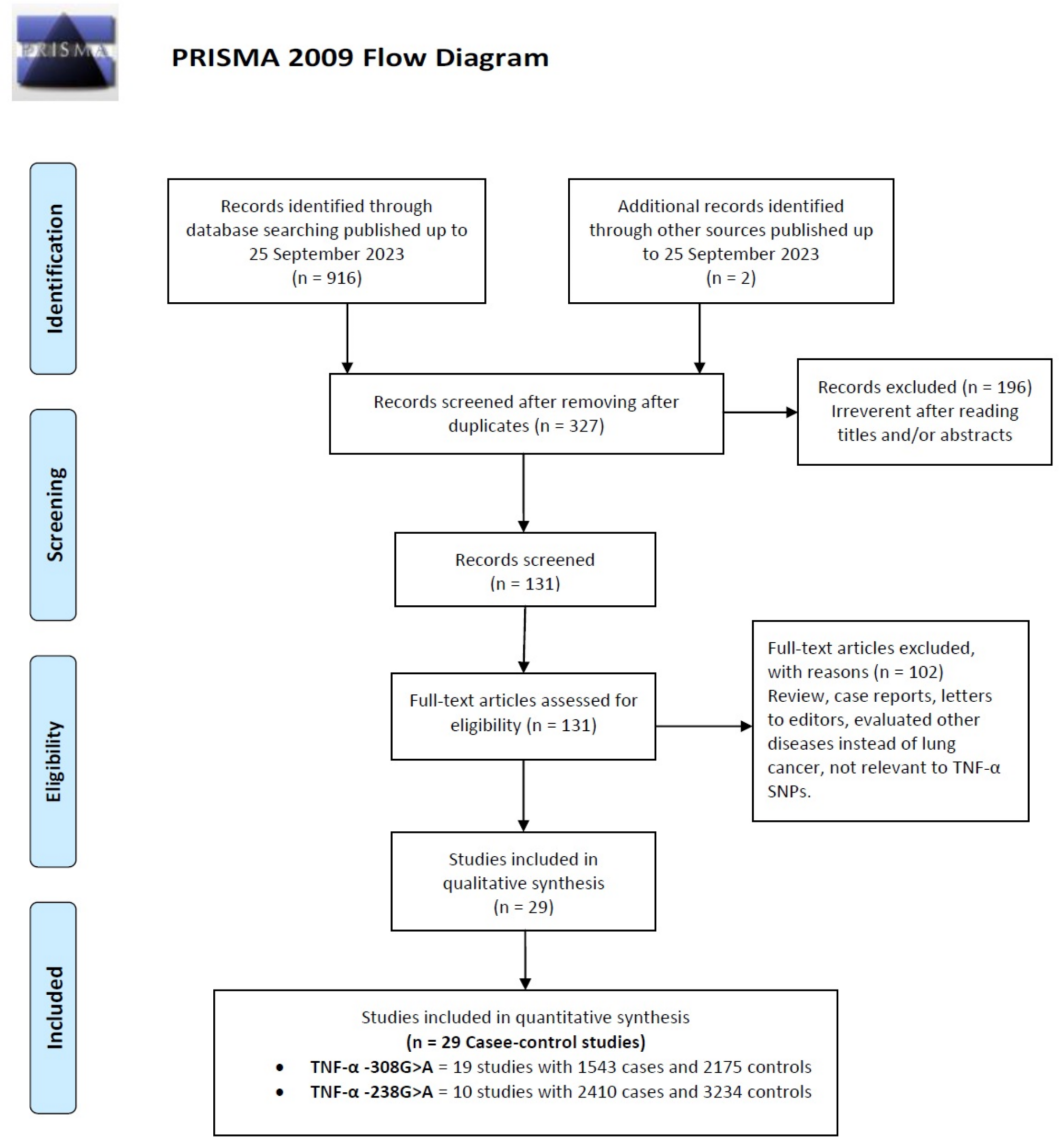
Flow diagram of study selection for the meta-analysis

Figure 1 indicates that 918 studies were initially identified. After screening titles and abstracts, 591 irrelevant articles were excluded, along with 298 others for reasons such as lack of controls, insufficient data, or focus on different polymorphic sites of the TNF-α gene. Ultimately, 29 case-control studies involving 6,265 lung cancer cases and 7,663 healthy subjects were analyzed to evaluate the association between TNF-α polymorphisms and lung cancer susceptibility. Among these, 19 studies from 12 publications [18–32] focused on the − 308G > A polymorphism (3,838 cases, 5,306 controls), while ten studies [20, 24, 25, 27, 28, 31–33] examined the − 238G > A polymorphism (2,427 cases, 2,357 controls). Table 1 presents the characteristics of these studies, which span from 2005 to 2021 and include significant representation from Asian populations (China, India, Tunisia) and Caucasian populations (Germany, Croatia, Turkey, Serbia, USA), with fewer African subjects from the USA and Tunisia. Sample sizes varied widely, from 29 to 617 for cases and 48 to 839 for controls, primarily utilizing PCR-based genotyping methods such as PCR-RFLP, SSP-PCR, and TaqMan assays. Notably, some studies reported overlapping populations for different polymorphisms, particularly the research by Flego in 2009 and 2013, which emphasized SCLC and NSCLC. The review included 16 HB and 5 PB studies.

**Table 1:** Characteristics of studies in this meta-analysis

First Author/Year	Country (Ethnicity)	Type	SOC	Genotyping Methods	Case/Control	Patients					Healthy Control					MAFs	HWE
						Genotypes			Alleles		Genotypes			Alleles			
-308G > A						**GG**	**GA**	**AA**	**G**	**A**	**GG**	**GA**	**AA**	**G**	**A**		
Seifart 2005	Germany(Caucasian)	SCLC	PB	PCR-RFLP	40/242	29	11	0	69	11	171	67	4	409	75	0.155	0.373
Seifart 2005	Germany(Caucasian)	NSCLC	PB	PCR-RFLP	77/242	51	25	1	127	27	171	67	4	409	75	0.155	0.373
Huang 2005	China(Asian)	NSCLC	HB	PCR-RFLP	200/205	55	10	0	120	10	64	1	0	129	1	0.008	0.950
Shih 2006	China(Asian)	NSCLC	PB	PCR-RFLP	65/65	110	75	15	295	105	169	34	2	372	38	0.093	0.842
Colakogullari 2008	Turkey(Caucasian)	NSCLC	HB	SSP-PCR	33/59	28	5	0	61	5	37	16	6	90	28	0.237	0.054
Stankovic 2009	Serbia(Caucasian)	NSCLC	HB	PCR-RFLP	70,102	57	13	0	127	13	71	28	3	170	34	0.167	0.905
Van Dyke 2009	USA(African)	NSCLC	HB	GoldenGate	94/102	79	15	0	173	15	74	27	1	175	29	0.142	0.388
Van Dyke 2009	USA(Caucasian)	NSCLC	HB	GoldenGate	354/374	260	87	7	607	101	254	110	10	618	130	0.174	0.640
Flego 2009	Croatia(Caucasian)	SCLC	HB	PCR-RFLP	29/230	23	5	1	51	7	171	53	6	395	65	0.141	0.444
Flego 2009	Croatia(Caucasian)	NSCLC	HB	PCR-RFLP	201/230	146	47	8	339	63	171	53	6	395	65	0.141	0.444
Helmig 2010	Germany(Caucasian)	LC	HB	Rapid capillary PCR	374/177	290	79	5	659	89	136	38	3	310	44	0.124	0.854
Oh 2010	USA(Caucasian)	LC	PB	SNPlex assay	75/839	56	18	1	130	20	632	194	13	1458	220	0.131	0.666
Flego 2013	Croatia(Caucasian)	SCLC	HB	PCR-RFLP	33/230	27	5	1	59	7	171	53	6	395	65	0.141	0.444
Flego 2013	Croatia(Caucasian)	NSCLC	HB	PCR-RFLP	272/230	192	67	13	451	93	171	53	6	395	65	0.141	0.444
Kaabachi 2013	Tunisia(African)	NSCLC	HB	PCR-RFLP	133/174	73	50	10	196	70	142	29	3	313	35	0.101	0.298
Peddireddy 2016	India(Asian)	NSCLC	PB	ARMS-PCR	246/250	19	209	18	247	245	14	221	15	249	251	0.502	≤ 0.001
Tian 2016	China(Asian)	LC	NA	RT-PCR	500/500	441	43	16	925	75	354	105	41	813	187	0.187	≤ 0.001
Eaton 2018	USA(Caucasian)	LC	NA	TaqMan	617/617	409	181	27	999	235	404	193	20	1001	233	0.189	0.599
Jia 2021	China(Asian)	NSCLC	NA	TaqMan	425/438	390	35	0	815	35	394	43	1	831	45	0.051	0.878
-238G > A						**GG**	**GA**	**AA**	**G**	**A**	**GG**	**GA**	**AA**	**G**	**A**		
Shih 2006	China(Asian)	NSCLC	PB	PCR-RFLP	202/205	187	15	0	389	15	161	44	0	366	44	0.107	0.085
Flego 2009	Croatia(Caucasian)	SCLC	HB	PCR-RFLP	29/230	27	2	0	56	2	214	16	0	444	16	0.035	0.584
Flego 2009	Croatia(Caucasian)	NSCLC	HB	PCR-RFLP	201/230	189	12	0	390	12	214	16	0	444	16	0.035	0.584
Helmig 2010	Germany(Caucasian)	LC	HB	Rapid capillary PCR	374/177	338	36	0	712	36	162	15	0	339	15	0.042	0.556
Flego 2013	Croatia(Caucasian)	SCLC	HB	PCR-RFLP	33/48	31	2	0	64	2	23	2	23	48	48	0.500	≤ 0.001
Flego 2013	Croatia(Caucasian)	NSCLC	HB	PCR-RFLP	272/98	260	12	0	532	12	98	0	0	196	0	NA	NA
Liang 2013	China(Asian)	LC	NA	PCR-RFLP	138/138	99	25	14	223	53	113	23	2	249	27	0.098	0.512
Kaabachi 2013	Tunisia(African)	NSCLC	HB	PCR-RFLP	132/172	80	38	14	198	66	130	40	2	300	44	0.128	0.577
Eaton 2018	USA(Caucasian)	LC	NA	TaqMan	621/621	556	64	1	1176	66	561	58	2	1180	62	0.050	0.701
Jia 2021	China(Asian)	NSCLC	NA	TaqMan	425/438	410	15	0	835	15	421	17	0	859	17	0.012	0.678

**Abbreviations**: LC - lung cancer; SCLC - small cell lung cancer; NSCLC - non-small cell lung cancer; HB - hospital-based; PB - population-based; RFLP - PCR-restriction fragment length polymorphism; SSP-PCR - sequence-specific primers; ARMS-PCR - amplification refractory mutation system polymerase chain; RT-PCR - real-time PCR; MAF - minor allele frequency; HWE - Hardy-Weinberg equilibrium.

### Quality of studies included

The meta-analysis evaluation indicates an overall improvement in study quality over time, especially from 2013 onwards. Earlier studies (2005–2010) had moderate sample sizes and primarily used PCR-RFLP for genotyping, with a mix of PB and HB controls, which occasionally introduced bias. In contrast, more recent studies (2013–2021) featured larger sample sizes and employed modern genotyping techniques like TaqMan, enhancing the reliability of their findings. These studies also demonstrated a broader approach to control sourcing and generally exhibited better compliance with Hardy-Weinberg equilibrium, reflecting improved methodological rigor. The variation in minor allele frequencies and compliance with HWE highlights the diverse genetic backgrounds of the studied populations. Overall, studies combining both PB and HB controls with advanced genotyping methods are recognized for their robustness, contributing to more valid and generalizable conclusions in the meta-analysis.

### Quantitative data synthesis

#### -308G > A

**Fig. 2:**
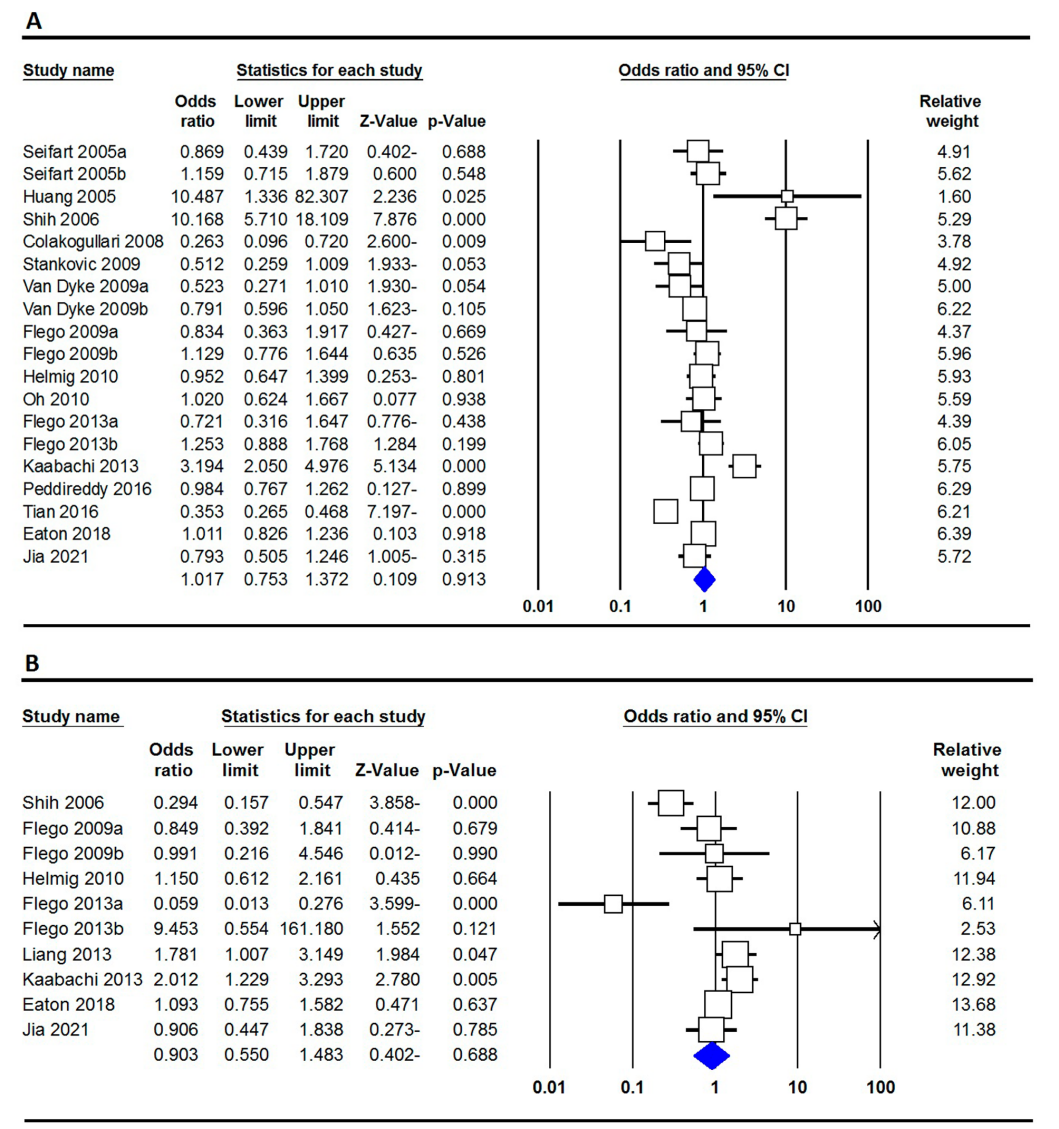
Forest plot depicting the correlation of TNF-α -308G > A and − 238G > A polymorphisms with lung cancer risk: (**A**) allele model (A vs. G); (**B**) dominant model (AA + AG vs. GG)

The meta-analysis of the TNF-α -308G > A polymorphism and lung cancer risk, as shown in Table 2, revealed varied associations based on genetic models and populations. Most comparisons indicated no significant links, with fixed ORs suggesting neutrality for A vs. G (0.953), AA vs. GG (0.947), AA + AG vs. GG (0.938), and AA vs. AG + GG (1.019). However, the AG vs. GG comparison showed a significant reduction in lung cancer risk with a fixed OR of 0.868 (*p* = 0.017). Figure 2A displays a forest plot showing the relationship between the TNF-α -308G > A polymorphism and lung cancer risk under the allele model. In the Asian subgroup, significant associations emerged, with reduced risk evident in A vs. G (fixed OR: 0.831, *p* = 0.028) and AA vs. GG (fixed OR: 0.571, *p* = 0.021). Conversely, the Caucasian subgroup found no significant associations. For NSCLC, significant associations were noted in A vs. G (fixed OR: 1.131, *p* = 0.049) and a trend in AA + AG vs. GG (fixed OR: 1.154, *p* = 0.060), while SCLC showed no significant links.

**Table 2:** Meta-analysis results of the TNF-α -308G > A polymorphism and lung cancer risk

Genetic Model	Type of Model	Heterogeneity		Odds ratio				Publication Bias	
		I^2^(%)	*P*_H_	OR	95% CI	Z_OR_	*P*_OR_	*P*_Beggs_	*P*_Eggers_
Overall									
A vs. G	Fixed	89.12	≤ 0.001	0.953	0.870–1.045	-1.025	0.305	0.528	0.544
	Random			1.017	0.753–1.372	0.109	0.913		
AA vs. GG	Fixed	58.40	0.001	0.947	0.714–1.256	-0.377	0.706	0.197	0.816
	Random			1.054	0.629–1.765	0.200	0.841		
AG vs. GG	Fixed	74.43	≤ 0.001	0.868	0.773–0.975	-2.380	0.017	0.704	0.738
	Random			0.880	0.683–1.135	-0.984	0.325		
AA + AG vs. GG	Fixed	85.55	≤ 0.001	0.938	0.842–1.046	-1.154	0.248	0.528	0.739
	Random			0.957	0.704–1.303	-0.277	0.782		
AA vs. AG + GG	Fixed	48.05	0.012	1.019	0.777–1.337	0.138	0.891	0.129	0.968
	Random			1.101	0.705–1.718	0.423	0.672		
Caucasian									
A vs. G	Fixed	31.27	0.141	0.957	0.853–1.073	-0.750	0.454	0.004	0.023
	Random			0.937	0.803–1.093	-0.833	0.405		
AA vs. GG	Fixed	0.00	0.765	1.118	0.775–1.613	0.596	0.551	0.011	0.007
AG vs. GG	Fixed	0.00	0.567	0.927	0.802–1.072	-1.017	0.309	0.114	0.074
AA + AG vs. GG	Fixed	9.756	0.350	0.931	0.817–1.061	-1.069	0.285	0.114	0.164
	Random			0.928	0.804–1.071	-1.024	0.306		
AA vs. AG + GG	Fixed	0.00	0.858	1.208	0.818–1.783	0.948	0.343	0.007	0.006
Asian									
A vs. G	Fixed	96.52	≤ 0.001	0.831	0.704–0.980	-2.204	0.028	1.000	0.713
	Random			1.621	0.585–4.487	0.930	0.353		
AA vs. GG	Fixed	84.80	≤ 0.001	0.571	0.355–0.920	-2.305	0.021	0.734	0.432
	Random			1.065	0.225–5.041	0.079	0.937		
AG vs. GG	Fixed	83.80	≤ 0.001	0.529	0.404–0.694	-4.604	≤ 0.001	0.308	0.182
	Random			0.773	0.348–1.720	-0.630	0.529		
AA + AG vs. GG	Fixed	95.01	≤ 0.001	0.794	0.637–0.989	-2.055	0.040	0.806	0.445
	Random			1.262	0.419–3.804	0.414	0.679		
AA vs. AG + GG	Fixed	83.58	≤ 0.001	0.749	0.487–1.154	-1.311	0.190	0.734	0.552
	Random			1.170	0.313–4.371	0.233	0.816		
NSCLC									
A vs. G	Fixed	89.98	≤ 0.001	1.131	1.001–1.277	1.970	0.049	0.755	0.481
	Random			1.291	0.835–1.997	1.150	0.250		
AA vs. GG	Fixed	55.85	0.012	1.412	0.925–2.154	1.601	0.109	0.755	0.356
	Random			1.303	0.641–2.648	0.731	0.465		
AG vs. GG	Fixed	73.36	≤ 0.001	0.984	0.837–1.157	-0.194	0.846	0.876	0.784
	Random			0.985	0.700-1.386	-0.086	0.932		
AA + AG vs. GG	Fixed	85.74	≤ 0.001	1.154	0.994–1.339	1.881	0.060	0.631	0.936
	Random			1.110	0.728–1.693	0.487	0.626		
AA vs. AG + GG	Fixed	42.62	0.065	1.420	0.961–2.097	1.762	0.078	0.533	0.357
	Random			1.381	0.769–2.479	1.080	0.280		
SCLC									
A vs. G	Fixed	0.00	0.492	1.118	0.838–1.492	0.757	0.449	1.000	0.097
AA vs. GG	Fixed	0.00	0.940	1.013	0.261–3.930	0.019	0.985	1.000	0.193
AG vs. GG	Fixed	0.00	0.729	0.783	0.467–1.313	-0.926	0.355	1.000	0.200
AA + AG vs. GG	Fixed	0.00	0.845	0.785	0.478–1.290	-0.954	0.340	1.000	0.299
AA vs. AG + GG	Fixed	0.00	0.926	1.088	0.282–4.203	0.123	0.902	1.000	0.144

#### -238G > A

The TNF-α -238G > A polymorphism presents a complex association with lung cancer risk, as detailed in Table 3. While the A allele shows a slight, non-significant risk increase (OR: 1.144, CI: 0.946–1.383), individuals with the AA genotype have a significant risk when compared to GG (OR: 3.171, CI: 1.261–7.970, *p* = 0.014). The AG genotype does not significantly differ from GG (OR: 0.992), nor does the combined AA + AG when compared to GG (OR: 1.047). However, the AA genotype significantly increases risk compared to AG + GG (OR: 3.003, CI: 1.198–7.528, *p* = 0.019). Figure 2B presents a forest plot indicating the association between the TNF-α -238G > A polymorphism and lung cancer risk in the dominant model. Population-specific analyses reveal further nuances: in the Caucasian subgroup, AA shows a protective effect (OR: 0.120, *p* = 0.024), while in the Asian subgroup, AA is associated with a high risk (OR: 7.990, *p* = 0.007) and AG with a reduced risk (OR: 0.674, *p* = 0.039). For SCLC, there are protective effects for A vs. G (OR: 0.422, *p* = 0.012) and AA vs. GG (OR: 0.016, *p* = 0.004), while NSCLC shows a significant risk increase for AA vs. GG (OR: 11.375, *p* = 0.002). Overall, the TNF-α -238G > A polymorphism highlights variations in lung cancer risk across different populations and cancer types, revealing both risk and protective effects based on genotype.

**Table 3:** Meta-analysis results for the TNF-α -238G > A polymorphism and lung cancer risk

Genetic Model	Type of Model	Heterogeneity		Odds ratio				Publication Bias	
		I^2^(%)	*P*_H_	OR	95% CI	Z_OR_	*P*_OR_	*P*_Beggs_	*P*_Eggers_
Overall									
A vs. G	Fixed	85.08	≤ 0.001	1.144	0.946–1.383	1.385	0.166	0.474	0.336
	Random			0.893	0.514–1.551	-0.402	0.688		
AA vs. GG	Fixed	84.77	≤ 0.001	3.171	1.261–7.970	2.454	0.014	0.308	0.021
	Random			1.183	0.097–14.434	0.132	0.895		
AG vs. GG	Fixed	57.57	0.012	0.992	0.805–1.223	-0.074	0.941	0.591	0.927
	Random			0.966	0.672–1.389	-0.185	0.853		
AA + AG vs. GG	Fixed	78.76	≤ 0.001	1.047	0.854–1.282	0.440	0.660	0.371	0.492
	Random			0.903	0.550–1.483	-0.402	0.688		
AA vs. AG + GG	Fixed	84.26	≤ 0.001	3.003	1.198–7.528	2.346	0.019	0.089	0.021
	Random			1.145	0.098–13.331	0.108	0.914		
Caucasian									
A vs. G	Fixed	79.36	≤ 0.001	0.945	0.719–1.244	-0.402	0.688	1.000	0.579
	Random			0.720	0.326–1.590	-0.812	0.417		
AA vs. GG	Fixed	69.74	0.069	0.120	0.019–0.754	-2.262	0.024	NA	NA
	Random			0.98	0.003–2.887	-1.346	0.178		
AG vs. GG	Fixed	0.00	0.728	1.090	0.818–1.453	0.590	0.555	1.000	0.595
	Random			1.090	0.818–1.453	0.590	0.555		
AA + AG vs. GG	Fixed	68.62	0.007	0.985	0.742–1.307	-0.105	0.916	0.707	0.629
	Random			0.820	0.422–1.592	-0.587	0.557		
AA vs. AG + GG	Fixed	69.24	0.071	0.120	0.019–0.753	-2.263	0.024	NA	NA
	Random			0.098	0.003–2.815	-1.135	0.175		
Asian									
A vs. G	Fixed	91.40	≤ 0.001	0.083	0.702–1.376	-0.100	0.921	1.000	0.589
	Random			0.868	0.269–2.807	-0.236	0.814		
AA vs. GG	Fixed	0.00	1.000	7.990	1.772–36.022	2.705	0.007	NA	NA
	Random			7.990	1.772–36.022	2.705	0.007		
AG vs. GG	Fixed	82.06	0.004	0.674	0.463–0.980	-2.064	0.039	1.000	0.779
	Random			0.688	0.283–1.672	-0.826	0.409		
AA + AG vs. GG	Fixed	88.66	≤ 0.001	0.813	0.567–1.167	-1.121	0.262	1.000	0.782
	Random			0.782	0.265–2.309	-0.445	0.656		
AA vs. AG + GG	Fixed	0.00	1.000	7.677	1.711–34.458	2.661	0.008	NA	NA
	Random			7.677	1.711–34.458	2.661	0.008		
SCLC									
A vs. G	Fixed	93.53	≤ 0.001	0.422	0.215–0.829	-2.503	0.012	NA	NA
	Random			0.175	0.006–5.189	-1.007	0.314		
AA vs. GG	Fixed	0.00	1.000	0.016	0.001–0.275	-2.848	0.004	NA	NA
AG vs. GG	Fixed	0.00	0.823	0.893	0.264–3.022	-0.182	0.855	NA	NA
AA + AG vs. GG	Fixed	89.10	0.002	0.496	0.249–0.990	-1.987	0.047	NA	NA
	Random			0.243	0.018–3.302	-1.060	0.289		
AA vs. AG + GG	Fixed	0.00	1.000	0.016	0.001–0.279	-2.837	0.005	NA	NA
NSCLC									
A vs. G	Fixed	86.57	≤ 0.001	1.154	1.560–0.928	0.928	0.353	1.000	0.855
	Random			1.102	0.405–2.992	0.190	0.850		
AA vs. GG	Fixed	0.00	1.000	11.375	2.519–51.365	3.161	0.002	NA	NA
AG vs. GG	Fixed	78.96	0.001	0.843	0.601–1.181	-0.994	0.320	1.000	0.682
	Random			0.935	0.395–2.214	-0.153	0.770		
AA + AG vs. GG	Fixed	84.05	≤ 0.001	0.978	0.704–1.359	-0.132	0.895	1.000	0.887
	Random			1.036	0.393–2.731	0.071	0.965		
AA vs. AG + GG	Fixed	0.00	1.000	10.085	2.250-45.201	3.20	0.003	NA	NA

NA: Not Applicable

### Sensitivity analysis

Sensitivity analysis evaluated the stability of the link between TNF-α polymorphisms and lung cancer susceptibility, showing that both overall and stratified outcomes remained statistically robust despite variations in study inclusion. Excluding studies that did not comply with HWE further highlighted the consistency of the findings and mitigated potential biases related to sample selection and genotyping. Despite challenges from diverse methodologies, the analysis confirmed a stable association between TNF-α polymorphisms and lung cancer susceptibility. These results indicate a genuine link that warrants further investigation into the mechanisms connecting TNF-α to lung cancer, underscoring the need for rigorous methodological standards in genetic epidemiological research and continued exploration in this field.

### Heterogeneity test

The evaluation of TNF-α polymorphisms and their association with lung cancer susceptibility showed significant heterogeneity for the TNF-α -308G > A polymorphism. I2 values varied between 48.05% and 89.12%, with p-values for heterogeneity (PH) ranging from ≤ 0.001 to 0.012. Subgroup analyses revealed low to moderate heterogeneity in the Caucasian population (I2: 0–36.94%, PH: 0.095 to 0.858), while the Asian subgroup exhibited very high heterogeneity (I2: 83.58–96.52%, PH ≤ 0.001). For lung cancer subtypes, the SCLC subgroup had low heterogeneity (I2: 0–0.00%; PH: 0.845 to 0.940), whereas the NSCLC subgroup showed high heterogeneity (I2: 42.62–90.41%; PH: ≤0.001 to 0.065). In contrast, the TNF-α -238G > A polymorphism exhibited low heterogeneity overall, with I2 values from 0 to 8.84% and PH values between 0.357 and 0.895, indicating minimal variability among studies.

### Publication bias

**Fig. 3:**
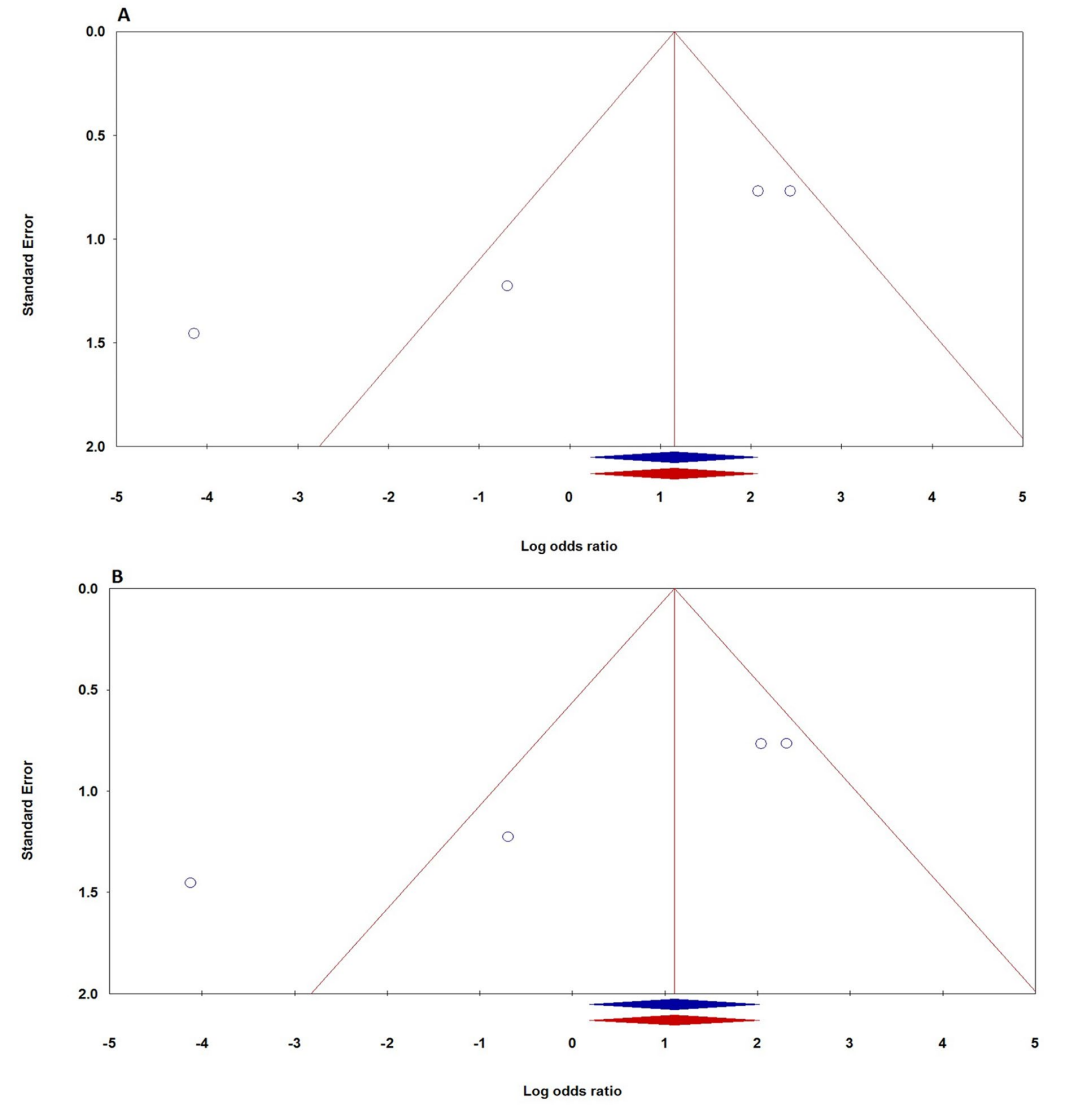
Begg’s funnel plot assessing publication bias for the correlation between TNF-α -238G > A polymorphism and lung cancer risk: (**A**) homozygote model (AA vs. GG); (**B**) recessive model (AA vs. AG + GG)

The assessment of publication bias regarding the TNF-α -308G > A polymorphism showed notable results, especially in the Caucasian subgroup, where Begg’s test indicated potential bias, with PBeggs values between 0.007 and 0.114, and Egger’s test results ranging from 0.006 to 0.074, both below the 0.05 significance threshold. No publication bias was found in the overall population or the Asian subgroup, with PBeggs at 1.000 and PEggers between 0.182 and 0.713. Analyses of SCLC and NSCLC also indicated no evidence of publication bias, with SCLC showing PBeggs at 1.000 and PEggers ranging from 0.144 to 0.299, and NSCLC having PBeggs from 0.533 to 0.876 and PEggers from 0.356 to 0.936. Similarly, the TNF-α -238G > A polymorphism showed no evidence of publication bias in subgroup analyses, with Begg’s p-values between 0.174 and 1.000 and Egger’s p-values from 0.089 to 0.893, all above 0.05. However, significant publication bias was detected in the overall population using homozygote and recessive models, indicated by Begg’s p-values of 0.308 and 0.089, and an Egger’s p-value of 0.021. To address this bias, the “trim and fill” method by Duval and Tweedie was employed, and comparisons of outcomes with and without this adjustment showed no significant differences, reinforcing the meta-analysis’s statistical robustness. Figure 3 displays Begg’s funnel plot assessing publication bias for the correlation between the TNF-α -238G > A polymorphism and lung cancer risk under both the homozygote model (AA vs. GG) and the recessive model (AA vs. AG + GG).

### HWE

The evaluation of HWE for various TNF-α polymorphisms shows significant variability influenced by factors such as geography, control source, genotyping methods, and cancer type. For instance, the − 308G > A polymorphism exhibited HWE values from ≤ 0.001 to 0.950, while the − 238G > A polymorphism ranged from ≤ 0.001 to 1.000. In Germany, HWE values were 0.373 for both SCLC and NSCLC for − 308G > A, and 0.556 for lung cancer (LC) with − 238G > A. In China, the HWE values for − 308G > A were higher at 0.950 and 0.842 (both NSCLC), while − 238G > A yielded 0.085 (NSCLC) and 0.512 (LC). Turkey reported an HWE of 0.054 (NSCLC) for − 308G > A and 0.577 (NSCLC) for − 238G > A. In the United States, HWE values were 0.640 for − 308G > A and 0.701 for − 238G > A among Caucasians, with Africans showing 0.388 for − 308G > A. Croatia had HWE values of 0.444 for SCLC and NSCLC with − 308G > A and 0.584 for both cancer types with − 238G > A. Tunisia reported 0.298 (NSCLC) for − 308G > A and 0.577 (NSCLC) for − 238G > A, while India had ≤ 0.001 for − 308G > A (NSCLC). Significant differences in HWE values were noted based on the control source, with PB studies showing greater discrepancies than HB studies. The genotyping methods used, such as PCR-RFLP, GoldenGate, and TaqMan, resulted in varied HWE outcomes, indicating that the method does not solely dictate HWE status. Studies on SCLC exhibited greater deviations from HWE compared to NSCLC analyses, which showed higher equilibrium prevalence.

### MAFs

The analysis of MAFs for the − 308G > A and − 238G > A polymorphisms shows significant variability across populations and lung cancer types. For − 308G > A, MAFs were 0.155 in Germany and Turkey, but much lower in China at 0.008 (overall) and 0.093 (NSCLC). India had the highest MAF at 0.502, indicating greater genetic susceptibility to lung cancer. In the U.S., MAFs were 0.142 for African Americans and 0.174 for Caucasians. For the − 238G > A polymorphism, China had a MAF of 0.107 for NSCLC, while Croatia reported lower frequencies of 0.035 for both SCLC and NSCLC. These results suggest heightened vulnerability to lung cancer in India and Turkey, contrasting with potential protective factors in China, and indicate differing genetic predispositions for SCLC and NSCLC.

## Discussion

Lung cancer is a multifaceted condition influenced by both environmental and genetic factors that significantly contribute to its development [2, 34]. A meta-analysis acts as a statistical method to aggregate findings from various research studies on a specific topic, thereby enhancing the statistical power needed to resolve discrepancies in the literature [35–38]. To date, several aggregated articles published between 2011 and 2017 have examined the link between TNF-α -308G > A and − 238G > A polymorphisms and lung cancer risk. However, key studies were excluded from existing meta-analyses, undermining their credibility. This study aims to perform a comprehensive meta-analysis to resolve the ongoing debate regarding the relationship between these TNF-α polymorphisms and lung cancer risk.

Our analysis of 19 case-control studies involving 3,838 cases and 5,306 controls indicates that the TNF-α -308G > A polymorphism may affect lung cancer risk in a population-specific manner. In the Asian subgroup, the A allele is linked to reduced lung cancer risk, while no significant associations were found in the Caucasian subgroup. Additionally, NSCLC exhibited a potential risk association with the A allele, unlike SCLC. These results highlight the importance of considering ethnicity and cancer subtype in genetic cancer risk studies. Conversely, a meta-analysis by Liu et al. (2017) with 20 studies (4,865 cases and 6,329 controls) found the − 308G > A variant associated with an increased overall risk of squamous cell carcinoma (SCC) in lung and oral cancers but not in skin SCC, basal cell carcinoma (BCC), or melanoma [39]. Similarly, Xie et al. (2014) analyzed 12 case-control studies (2,436 cases and 2,573 controls) and found a significant link between this polymorphism and lung cancer susceptibility, particularly among Asians, irrespective of tumor type (SCLC or NSCLC) [40]. In another study, Peng et al. (2012) evaluated the impact of eight polymorphisms, including TNF-α, IL-6, IL-1β, COX-2, and IL-10, on lung cancer risk across six studies (728 cases and 904 controls), showing that variants like TNF-α 308G > A did not significantly influence lung cancer susceptibility [41]. Overall, the evidence indicates that the TNF-α -308G > A polymorphism plays a complex role in lung cancer risk, driven by ethnicity and cancer subtype. It may reduce risk in Asians while showing no significant effect in Caucasians, and appears more relevant to NSCLC than SCLC. Some studies also indicate an increased risk of SCC linked to this variant in lung and oral cancers. These results emphasize the importance of analyzing genetic variations across specific populations and cancer types when evaluating lung cancer risk factors. Further research is necessary to clarify these relationships and improve risk stratification.

This meta-analysis of ten case-control studies, with 2,427 cancer cases and 2,357 controls, uncovers a complex relationship between the TNF-α -238G > A polymorphism and lung cancer risk, showing both risk and protective effects that vary by genotype and population. The AA genotype significantly increases risk in certain groups, particularly among Asians with NSCLC, while providing protection for Caucasians. The AG genotype appears neutral, and the A allele does not substantially raise overall risk. Supporting this, a recent meta-analysis by Pashapour et al. found a significant association between the TNF-α -238G > A polymorphism and heightened lung cancer risk across different populations [42]. Conversely, Zhou et al.‘s 2011 meta-analysis, encompassing 34 studies with 34,679 cancer cases and 41,186 healthy controls, found no significant link between this polymorphism and various cancers [43]. Overall, evidence indicates that the TNF-α -238G > A polymorphism plays a nuanced role in lung cancer risk influenced by genetic and demographic factors. The AA genotype correlates with increased risk for specific populations, such as Asians with NSCLC, while potentially protecting Caucasians. The AG genotype is neutral, and the impact of the A allele is minimal. These findings underscore the role of population genetics in cancer risk studies. Additionally, conflicting recent results highlight the necessity for careful study design and attention to demographics; targeted analyses may reveal ethnic differences overlooked in broader studies. Variations in statistical methods can account for these discrepancies, as newer studies might identify associations omitted in earlier research due to unadjusted confounders like age and smoking.

The investigation into TNF-α polymorphisms and lung cancer susceptibility reveals variability influenced by geographical, methodological, and demographic factors. For instance, significant differences in HWE values are seen among populations, with the − 308G > A polymorphism being more prevalent in China than in India, indicating the complexity of genetic susceptibility and environmental influences. Variations in control sources and genotyping methods also contribute to inconsistencies in HWE results. Assessments of MAFs highlight the need for consideration of population-specific genetic backgrounds; higher MAFs in India might indicate an increased lung cancer risk, while lower frequencies in China could signal protective factors. Additionally, analyses of heterogeneity reveal distinct patterns among ethnic groups and lung cancer subtypes, with Asian populations exhibiting greater heterogeneity for the − 308G > A polymorphism compared to Caucasians. This emphasizes the necessity of targeted genetic studies that reflect demographic diversity and methodological distinctions, ultimately improving strategies for understanding and addressing lung cancer susceptibility across populations.

The analysis of TNF-α -308G > A and − 238G > A polymorphisms in relation to lung cancer risk presents both strengths and weaknesses. On the positive side, it consolidates a variety of large-sample case-control studies, which enhances the validity of its conclusions. By highlighting ethnic and subtype variations, it clarifies the intricate genetic landscape of lung cancer, acknowledging the differing roles of these polymorphisms across populations. Additionally, the review identifies research gaps and emphasizes the need for further studies that account for confounding variables such as age and smoking. However, several limitations are noted, including small sample sizes in studies on the TNF-α -238G > A polymorphism, which diminish statistical power and the ability to establish a clear correlation with lung cancer risk. The focus on predominantly Asian and Caucasian patients restricts representation from African and mixed populations, hindering meaningful subgroup analyses. To enhance reliability and generalizability, future research should prioritize a more diverse range of ethnicities. Potential publication bias is another concern, as studies demonstrating positive correlations tend to be published more frequently, potentially skewing meta-analysis outcomes. The reliance on published literature primarily from English and Chinese databases may also overlook significant unpublished studies and those in other languages, contributing to systematic bias. Insufficient data for stratified analyses regarding confounding factors, including age, gender, smoking habits, and lung cancer types, limits the evaluation of their influence on TNF-α polymorphisms and lung cancer risk. Future research should aim to gather comprehensive data to address these factors adequately. Additionally, while lung cancer, like other cancers, is influenced by complex gene-environment interactions, primary studies often fail to address these adequately due to a lack of detailed information. Larger studies involving diverse populations should incorporate thorough data to clarify the roles of gene-gene and gene-environment interactions. Challenges persist, particularly regarding inconsistencies in study findings that raise questions about methodology and data reliability. Limited methodological insights hinder the assessment of study validity, risking overgeneralization of the impacts of environmental and lifestyle factors on genetic predispositions. Furthermore, inadequate exploration of clinical implications restricts the practical application of findings, while potential publication biases may distort the overall interpretations.

## Conclusions

The pooled analysis of TNF-α polymorphisms − 308G > A and − 238G > A reveals significant variability in lung cancer risk across populations and cancer types. While − 308G > A mainly shows neutral results, certain Asian subgroups exhibit notable risk reductions, suggesting a complex relationship between genetics and lung cancer susceptibility. In contrast, -238G > A is associated with a stronger risk, particularly for the AA genotype in Asians, while offering protective effects in Caucasians. The differences in small cell and NSCLC further underscore the need to consider genetic and environmental factors in cancer risk assessment. These findings emphasize the importance of personalized approaches to understanding lung cancer etiology and developing targeted prevention strategies.

## Data Availability

No datasets were generated or analysed during the current study.
